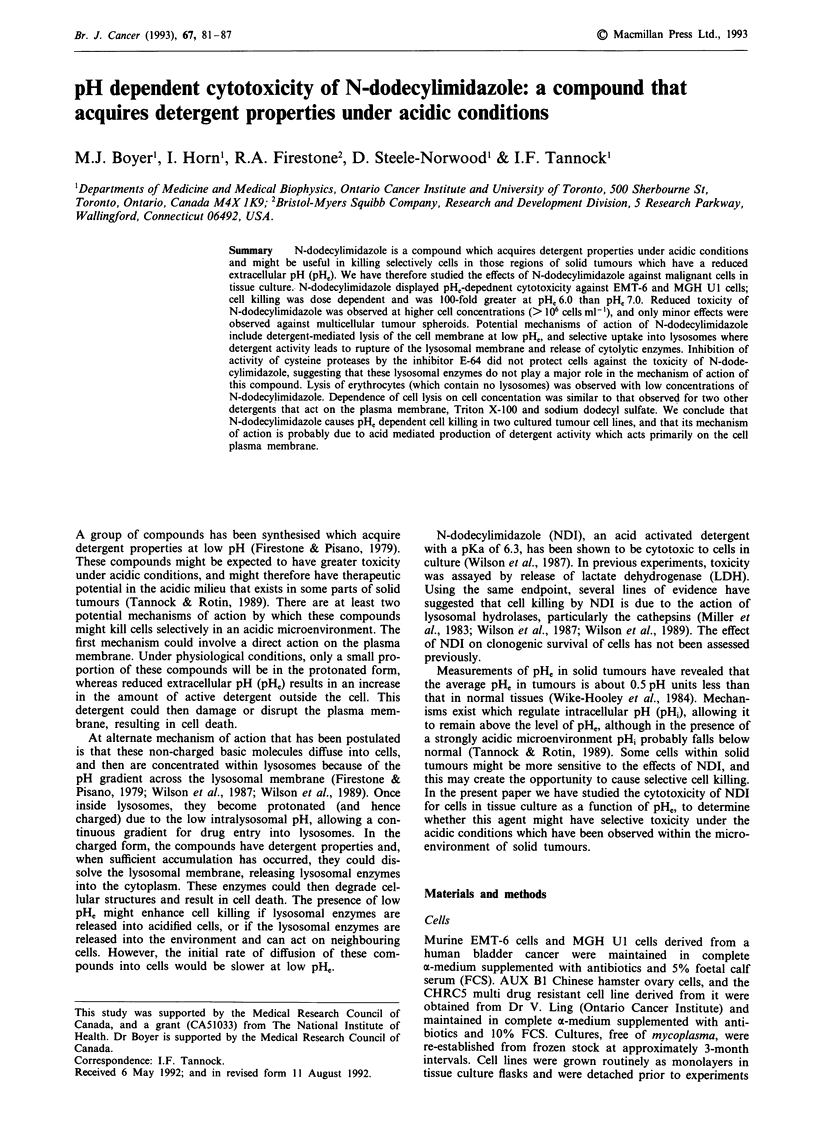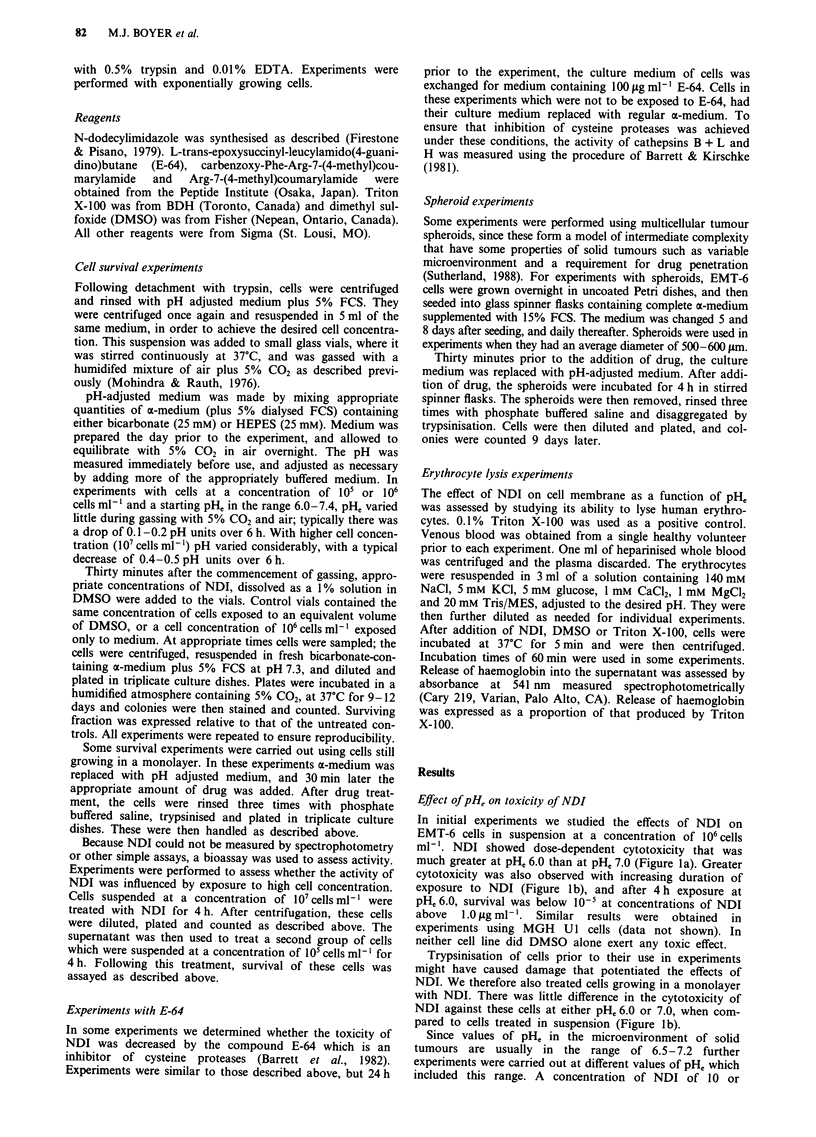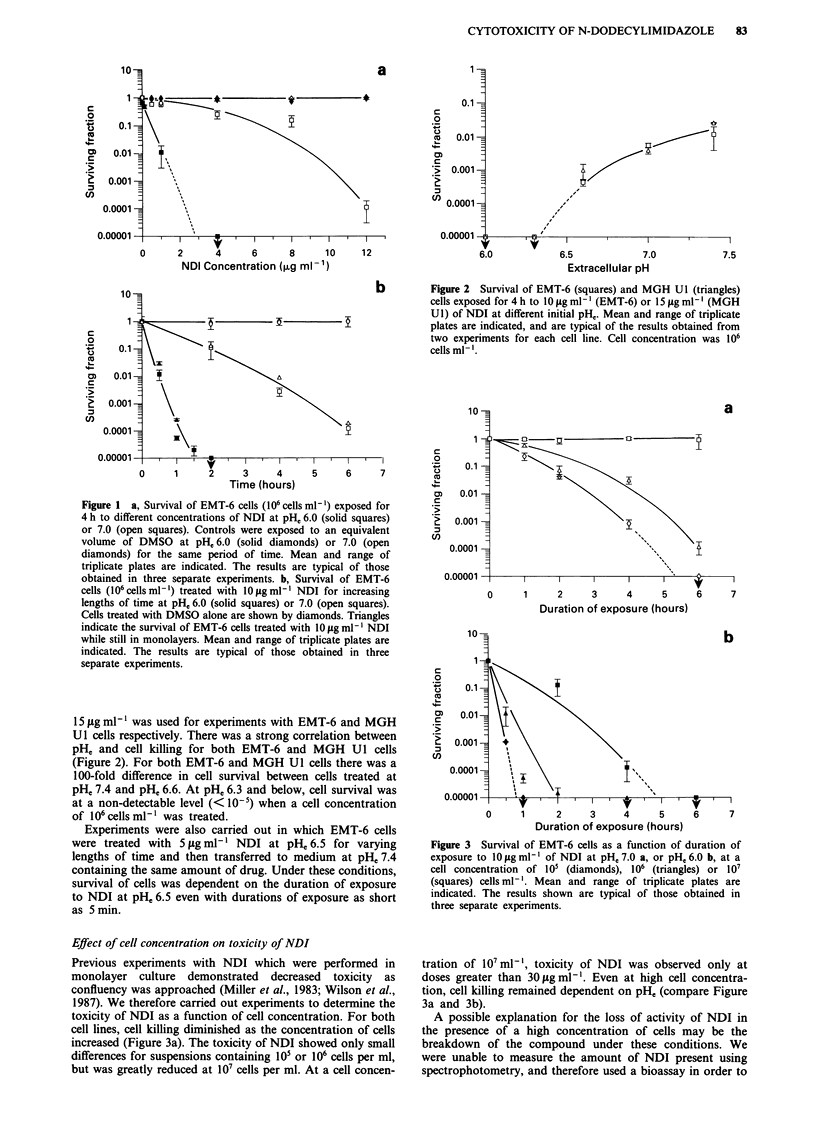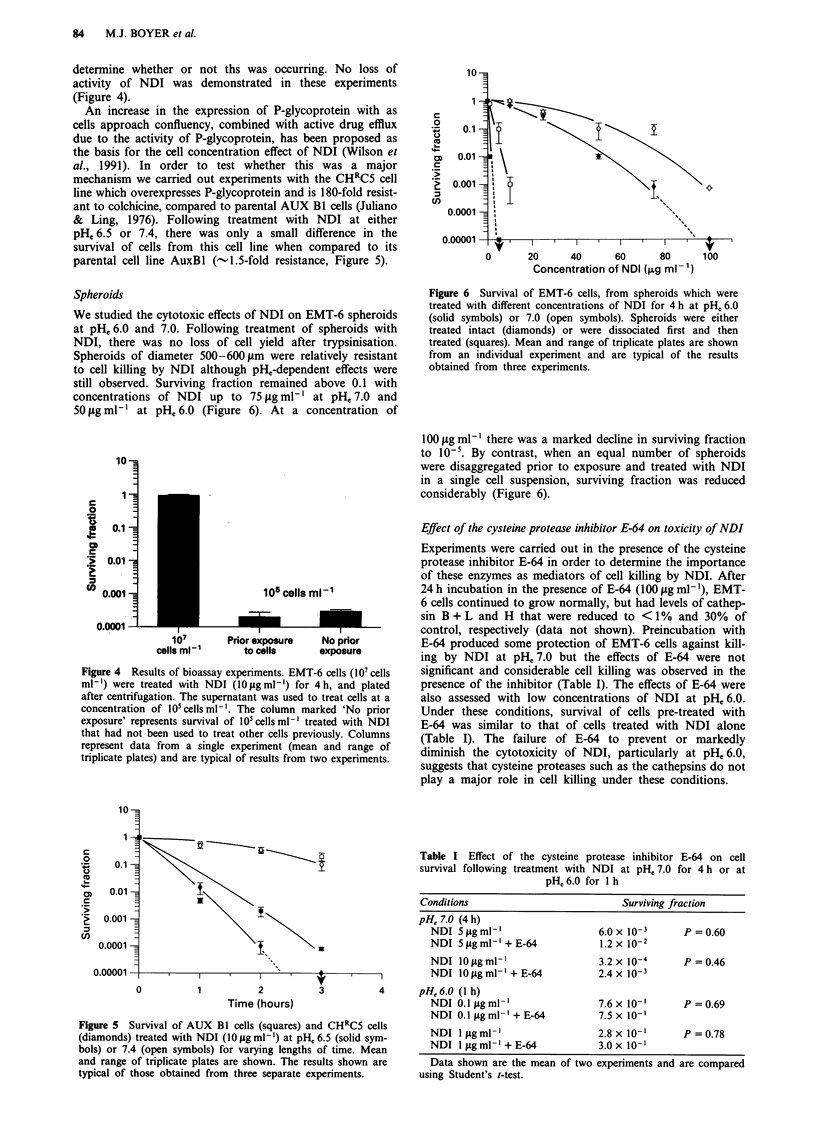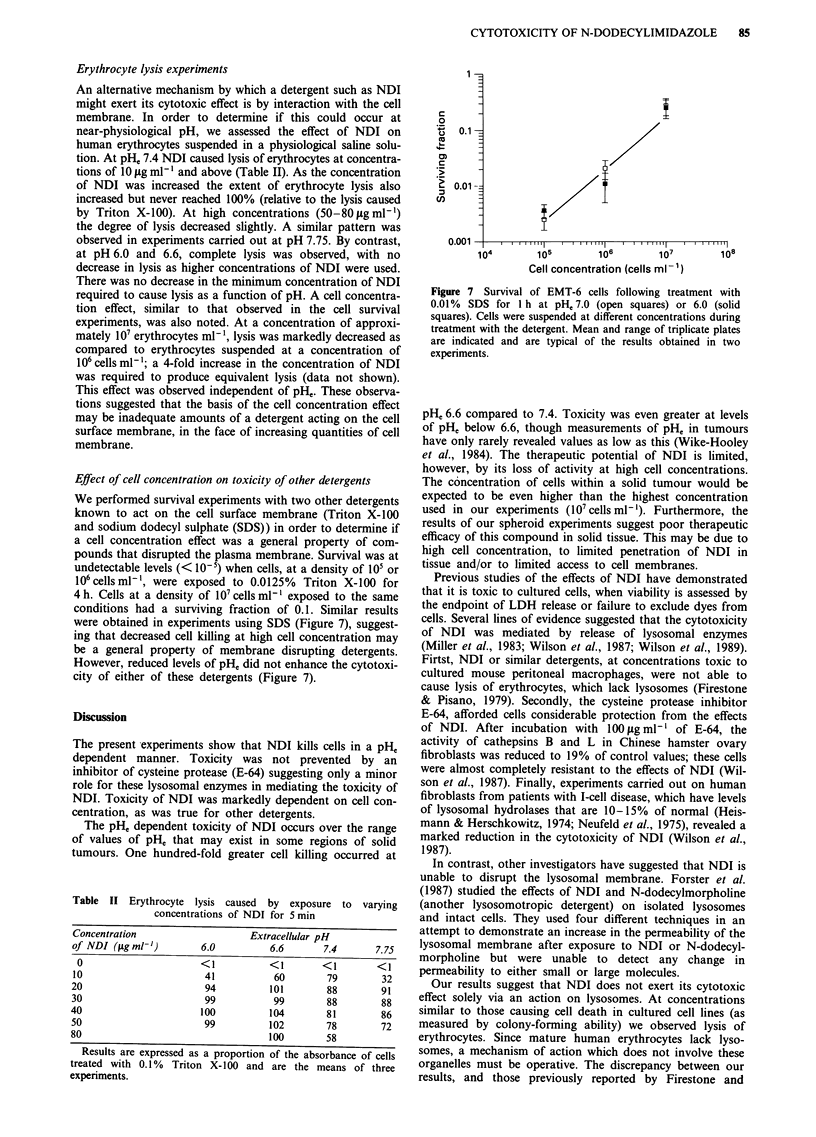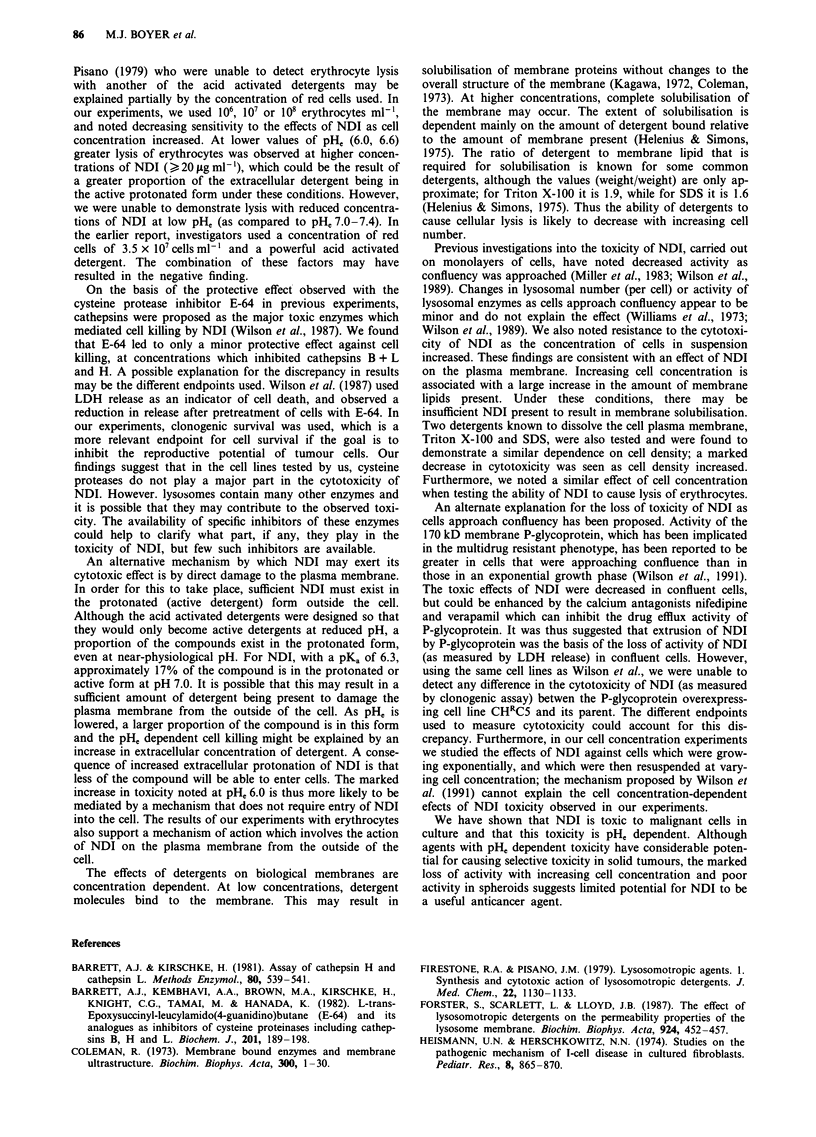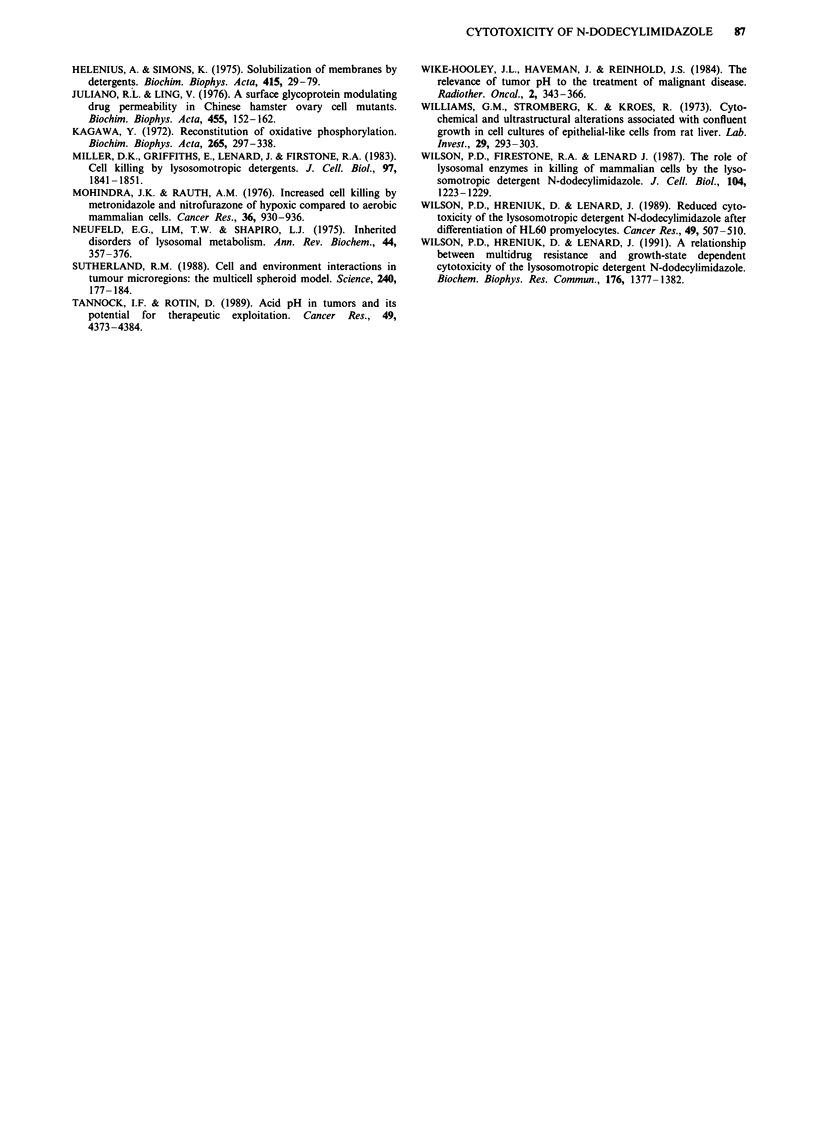# pH dependent cytotoxicity of N-dodecylimidazole: a compound that acquires detergent properties under acidic conditions.

**DOI:** 10.1038/bjc.1993.13

**Published:** 1993-01

**Authors:** M. J. Boyer, I. Horn, R. A. Firestone, D. Steele-Norwood, I. F. Tannock

**Affiliations:** Department of Medicine, Ontario Cancer Institute, Toronto, Canada.

## Abstract

N-dodecylimidazole is a compound which acquires detergent properties under acidic conditions and might be useful in killing selectively cells in those regions of solid tumours which have a reduced extracellular pH (pHe). We have therefore studied the effects of N-dodecylimidazole against malignant cells in tissue culture. N-dodecylimidazole displayed pHe-dependent cytotoxicity against EMT-6 and MGH U1 cells; cell killing was dose dependent and was 100-fold greater at pHe 6.0 than pHe 7.0. Reduced toxicity of N-dodecylimidazole was observed at higher cell concentrations (> 10(6) cells ml-1), and only minor effects were observed against multicellular tumour spheroids. Potential mechanisms of action of N-dodecylimidazole include detergent-mediated lysis of the cell membrane at low pHe, and selective uptake into lysosomes where detergent activity leads to rupture of the lysosomal membrane and release of cytolytic enzymes. Inhibition of activity of cysteine proteases by the inhibitor E-64 did not protect cells against the toxicity of N-dodecylimidazole, suggesting that these lysosomal enzymes do not play a major role in the mechanism of action of this compound. Lysis of erythrocytes (which contain no lysosomes) was observed with low concentrations of N-dodecylimidazole. Dependence of cell lysis on cell concentration was similar to that observed for two other detergents that act on the plasma membrane, Triton X-100 and sodium dodecyl sulfate. We conclude that N-dodecylimidazole causes pHe dependent cell killing in two cultured tumour cell lines, and that its mechanism of action is probably due to acid mediated production of detergent activity which acts primarily on the cell plasma membrane.


Br. J. Cancer (1993), 67, 81-87 © Macmillan Press Ltd., 1993

### pH dependent cytotoxicity of N-dodecylimidazole: a compound that acquires detergent properties under acidic conditions

M.J. Boyer[1], I. Horn[1], R.A. Firestone[2], D. Steele-Norwood[1] & I.F. Tannock[1]

[1]*Departments of Medicine and Medical Biophysics, Ontario Cancer Institute and University of Toronto, 500 Sherbourne St, Toronto, Ontario, Canada M4X 1K9;* [2]*Bristol-Myers Squibb Company, Research and Development Division, 5 Research Parkway, Wallingford, Connecticut 06492, USA.*

**Summary** N-dodecylimidazole is a compound which acquires detergent properties under acidic conditions and might be useful in killing selectively cells in those regions of solid tumours which have a reduced extracellular pH ($pH_e$). We have therefore studied the effects of N-dodecylimidazole against malignant cells in tissue culture. N-dodecylimidazole displayed $pH_e$-depednent cytotoxicity against EMT-6 and MGH U1 cells; cell killing was dose dependent and was 100-fold greater at $pH_e$ 6.0 than $pH_e$ 7.0. Reduced toxicity of N-dodecylimidazole was observed at higher cell concentrations (> $10^6$ cells $ml^{-1}$), and only minor effects were observed against multicellular tumour spheroids. Potential mechanisms of action of N-dodecylimidazole include detergent-mediated lysis of the cell membrane at low $pH_e$, and selective uptake into lysosomes where detergent activity leads to rupture of the lysosomal membrane and release of cytolytic enzymes. Inhibition of activity of cysteine proteases by the inhibitor E-64 did not protect cells against the toxicity of N-dodecylimidazole, suggesting that these lysosomal enzymes do not play a major role in the mechanism of action of this compound. Lysis of erythrocytes (which contain no lysosomes) was observed with low concentrations of N-dodecylimidazole. Dependence of cell lysis on cell concentation was similar to that observed for two other detergents that act on the plasma membrane, Triton X-100 and sodium dodecyl sulfate. We conclude that N-dodecylimidazole causes $pH_e$ dependent cell killing in two cultured tumour cell lines, and that its mechanism of action is probably due to acid mediated production of detergent activity which acts primarily on the cell plasma membrane.

A group of compounds has been synthesised which acquire detergent properties at low pH (Firestone & Pisano, 1979). These compounds might be expected to have greater toxicity under acidic conditions, and might therefore have therapeutic potential in the acidic milieu that exists in some parts of solid tumours (Tannock & Rotin, 1989). There are at least two potential mechanisms of action by which these compounds might kill cells selectively in an acidic microenvironment. The first mechanism could involve a direct action on the plasma membrane. Under physiological conditions, only a small proportion of these compounds will be in the protonated form, whereas reduced extracellular pH ($pH_e$) results in an increase in the amount of active detergent outside the cell. This detergent could then damage or disrupt the plasma membrane, resulting in cell death.

At alternate mechanism of action that has been postulated is that these non-charged basic molecules diffuse into cells, and then are concentrated within lysosomes because of the pH gradient across the lysosomal membrane (Firestone & Pisano, 1979; Wilson *et al.*, 1987; Wilson *et al.*, 1989). Once inside lysosomes, they become protonated (and hence charged) due to the low intralysosomal pH, allowing a continuous gradient for drug entry into lysosomes. In the charged form, the compounds have detergent properties and, when sufficient accumulation has occurred, they could dissolve the lysosomal membrane, releasing lysosomal enzymes into the cytoplasm. These enzymes could then degrade cellular structures and result in cell death. The presence of low $pH_e$ might enhance cell killing if lysosomal enzymes are released into acidified cells, or if the lysosomal enzymes are released into the environment and can act on neighbouring cells. However, the initial rate of diffusion of these compounds into cells would be slower at low $pH_e$.

This study was supported by the Medical Research Council of Canada, and a grant (CA51033) from The National Institute of Health. Dr Boyer is supported by the Medical Research Council of Canada.
Correspondence: I.F. Tannock.
Received 6 May 1992; and in revised form 11 August 1992.

N-dodecylimidazole (NDI), an acid activated detergent with a pKa of 6.3, has been shown to be cytotoxic to cells in culture (Wilson *et al.*, 1987). In previous experiments, toxicity was assayed by release of lactate dehydrogenase (LDH). Using the same endpoint, several lines of evidence have suggested that cell killing by NDI is due to the action of lysosomal hydrolases, particularly the cathepsins (Miller *et al.*, 1983; Wilson *et al.*, 1987; Wilson *et al.*, 1989). The effect of NDI on clonogenic survival of cells has not been assessed previously.

Measurements of $pH_e$ in solid tumours have revealed that the average $pH_e$ in tumours is about 0.5 pH units less than that in normal tissues (Wike-Hooley *et al.*, 1984). Mechanisms exist which regulate intracellular pH ($pH_i$), allowing it to remain above the level of $pH_e$, although in the presence of a strongly acidic microenvironment $pH_i$ probably falls below normal (Tannock & Rotin, 1989). Some cells within solid tumours might be more sensitive to the effects of NDI, and this may create the opportunity to cause selective cell killing. In the present paper we have studied the cytotoxicity of NDI for cells in tissue culture as a function of $pH_e$, to determine whether this agent might have selective toxicity under the acidic conditions which have been observed within the microenvironment of solid tumours.

### Materials and methods

### *Cells*

Murine EMT-6 cells and MGH U1 cells derived from a human bladder cancer were maintained in complete α-medium supplemented with antibiotics and 5% foetal calf serum (FCS). AUX B1 Chinese hamster ovary cells, and the CHRC5 multi drug resistant cell line derived from it were obtained from Dr V. Ling (Ontario Cancer Institute) and maintained in complete α-medium supplemented with antibiotics and 10% FCS. Cultures, free of *mycoplasma*, were re-established from frozen stock at approximately 3-month intervals. Cell lines were grown routinely as monolayers in tissue culture flasks and were detached prior to experiments with 0.5% trypsin and 0.01% EDTA. Experiments were performed with exponentially growing cells.

*Reagents*

N-dodecylimidazole was synthesised as described (Firestone & Pisano, 1979). L-trans-epoxysuccinyl-leucylamido(4-guanidino)butane (E-64), carbenzoxy-Phe-Arg-7-(4-methyl)coumarylamide and Arg-7-(4-methyl)coumarylamide were obtained from the Peptide Institute (Osaka, Japan). Triton X-100 was from BDH (Toronto, Canada) and dimethyl sulfoxide (DMSO) was from Fisher (Nepean, Ontario, Canada). All other reagents were from Sigma (St. Lousi, MO).

*Cell survival experiments*

Following detachment with trypsin, cells were centrifuged and rinsed with pH adjusted medium plus 5% FCS. They were centrifuged once again and resuspended in 5 ml of the same medium, in order to achieve the desired cell concentration. This suspension was added to small glass vials, where it was stirred continuously at 37°C, and was gassed with a humidifed mixture of air plus 5% $CO_2$ as described previously (Mohindra & Rauth, 1976).

pH-adjusted medium was made by mixing appropriate quantities of α-medium (plus 5% dialysed FCS) containing either bicarbonate (25 mM) or HEPES (25 mM). Medium was prepared the day prior to the experiment, and allowed to equilibrate with 5% $CO_2$ in air overnight. The pH was measured immediately before use, and adjusted as necessary by adding more of the appropriately buffered medium. In experiments with cells at a concentration of $10^5$ or $10^6$ cells $ml^{-1}$ and a starting $pH_e$ in the range 6.0–7.4, $pH_e$ varied little during gassing with 5% $CO_2$ and air; typically there was a drop of 0.1–0.2 pH units over 6 h. With higher cell concentration ($10^7$ cells $ml^{-1}$) pH varied considerably, with a typical decrease of 0.4–0.5 pH units over 6 h.

Thirty minutes after the commencement of gassing, appropriate concentrations of NDI, dissolved as a 1% solution in DMSO were added to the vials. Control vials contained the same concentration of cells exposed to an equivalent volume of DMSO, or a cell concentration of $10^6$ cells $ml^{-1}$ exposed only to medium. At appropriate times cells were sampled; the cells were centrifuged, resuspended in fresh bicarbonate-containing α-medium plus 5% FCS at pH 7.3, and diluted and plated in triplicate culture dishes. Plates were incubated in a humidified atmosphere containing 5% $CO_2$, at 37°C for 9–12 days and colonies were then stained and counted. Surviving fraction was expressed relative to that of the untreated controls. All experiments were repeated to ensure reproducibility.

Some survival experiments were carried out using cells still growing in a monolayer. In these experiments α-medium was replaced with pH adjusted medium, and 30 min later the appropriate amount of drug was added. After drug treatment, the cells were rinsed three times with phosphate buffered saline, trypsinised and plated in triplicate culture dishes. These were then handled as described above.

Because NDI could not be measured by spectrophotometry or other simple assays, a bioassay was used to assess activity. Experiments were performed to assess whether the activity of NDI was influenced by exposure to high cell concentration. Cells suspended at a concentration of $10^7$ cells $ml^{-1}$ were treated with NDI for 4 h. After centrifugation, these cells were diluted, plated and counted as described above. The supernatant was then used to treat a second group of cells which were suspended at a concentration of $10^5$ cells $ml^{-1}$ for 4 h. Following this treatment, survival of these cells was assayed as described above.

*Experiments with E-64*

In some experiments we determined whether the toxicity of NDI was decreased by the compound E-64 which is an inhibitor of cysteine proteases (Barrett *et al.*, 1982). Experiments were similar to those described above, but 24 h prior to the experiment, the culture medium of cells was exchanged for medium containing 100 $\mu g\ ml^{-1}$ E-64. Cells in these experiments which were not to be exposed to E-64, had their culture medium replaced with regular α-medium. To ensure that inhibition of cysteine proteases was achieved under these conditions, the activity of cathepsins B + L and H was measured using the procedure of Barrett & Kirschke (1981).

*Spheroid experiments*

Some experiments were performed using multicellular tumour spheroids, since these form a model of intermediate complexity that have some properties of solid tumours such as variable microenvironment and a requirement for drug penetration (Sutherland, 1988). For experiments with spheroids, EMT-6 cells were grown overnight in uncoated Petri dishes, and then seeded into glass spinner flasks containing complete α-medium supplemented with 15% FCS. The medium was changed 5 and 8 days after seeding, and daily thereafter. Spheroids were used in experiments when they had an average diameter of 500–600 μm.

Thirty minutes prior to the addition of drug, the culture medium was replaced with pH-adjusted medium. After addition of drug, the spheroids were incubated for 4 h in stirred spinner flasks. The spheroids were then removed, rinsed three times with phosphate buffered saline and disaggregated by trypsinisation. Cells were then diluted and plated, and colonies were counted 9 days later.

*Erythrocyte lysis experiments*

The effect of NDI on cell membrane as a function of $pH_e$ was assessed by studying its ability to lyse human erythrocytes. 0.1% Triton X-100 was used as a positive control. Venous blood was obtained from a single healthy volunteer prior to each experiment. One ml of heparinised whole blood was centrifuged and the plasma discarded. The erythrocytes were resuspended in 3 ml of a solution containing 140 mM NaCl, 5 mM KCl, 5 mM glucose, 1 mM $CaCl_2$, 1 mM $MgCl_2$ and 20 mM Tris/MES, adjusted to the desired pH. They were then further diluted as needed for individual experiments. After addition of NDI, DMSO or Triton X-100, cells were incubated at 37°C for 5 min and were then centrifuged. Incubation times of 60 min were used in some experiments. Release of haemoglobin into the supernatant was assessed by absorbance at 541 nm measured spectrophotometrically (Cary 219, Varian, Palo Alto, CA). Release of haemoglobin was expressed as a proportion of that produced by Triton X-100.

### Results

*Effect of $pH_e$ on toxicity of NDI*

In initial experiments we studied the effects of NDI on EMT-6 cells in suspension at a concentration of $10^6$ cells $ml^{-1}$. NDI showed dose-dependent cytotoxicity that was much greater at $pH_e$ 6.0 than at $pH_e$ 7.0 (Figure 1a). Greater cytotoxicity was also observed with increasing duration of exposure to NDI (Figure 1b), and after 4 h exposure at $pH_e$ 6.0, survival was below $10^{-5}$ at concentrations of NDI above 1.0 $\mu g\ ml^{-1}$. Similar results were obtained in experiments using MGH U1 cells (data not shown). In neither cell line did DMSO alone exert any toxic effect.

Trypsinisation of cells prior to their use in experiments might have caused damage that potentiated the effects of NDI. We therefore also treated cells growing in a monolayer with NDI. There was little difference in the cytotoxicity of NDI against these cells at either $pH_e$ 6.0 or 7.0, when compared to cells treated in suspension (Figure 1b).

Since values of $pH_e$ in the microenvironment of solid tumours are usually in the range of 6.5–7.2 further experiments were carried out at different values of $pH_e$ which included this range. A concentration of NDI of 10 or

**Figure 1** a, Survival of EMT-6 cells ($10^6$ cells $ml^{-1}$) exposed for 4 h to different concentrations of NDI at $pH_e$ 6.0 (solid squares) or 7.0 (open squares). Controls were exposed to an equivalent volume of DMSO at $pH_e$ 6.0 (solid diamonds) or 7.0 (open diamonds) for the same period of time. Mean and range of triplicate plates are indicated. The results are typical of those obtained in three separate experiments. b, Survival of EMT-6 cells ($10^6$ cells $ml^{-1}$) treated with 10 $\mu g\ ml^{-1}$ NDI for increasing lengths of time at $pH_e$ 6.0 (solid squares) or 7.0 (open squares). Cells treated with DMSO alone are shown by diamonds. Triangles indicate the survival of EMT-6 cells treated with 10 $\mu g\ ml^{-1}$ NDI while still in monolayers. Mean and range of triplicate plates are indicated. The results are typical of those obtained in three separate experiments.

**Figure 2** Survival of EMT-6 (squares) and MGH U1 (triangles) cells exposed for 4 h to 10 $\mu g\ ml^{-1}$ (EMT-6) or 15 $\mu g\ ml^{-1}$ (MGH U1) of NDI at different initial $pH_e$. Mean and range of triplicate plates are indicated, and are typical of the results obtained from two experiments for each cell line. Cell concentration was $10^6$ cells $ml^{-1}$.

15 $\mu g\ ml^{-1}$ was used for experiments with EMT-6 and MGH U1 cells respectively. There was a strong correlation between $pH_e$ and cell killing for both EMT-6 and MGH U1 cells (Figure 2). For both EMT-6 and MGH U1 cells there was a 100-fold difference in cell survival between cells treated at $pH_e$ 7.4 and $pH_e$ 6.6. At $pH_e$ 6.3 and below, cell survival was at a non-detectable level ($< 10^{-5}$) when a cell concentration of $10^6$ cells $ml^{-1}$ was treated.

Experiments were also carried out in which EMT-6 cells were treated with 5 $\mu g\ ml^{-1}$ NDI at $pH_e$ 6.5 for varying lengths of time and then transferred to medium at $pH_e$ 7.4 containing the same amount of drug. Under these conditions, survival of cells was dependent on the duration of exposure to NDI at $pH_e$ 6.5 even with durations of exposure as short as 5 min.

**Figure 3** Survival of EMT-6 cells as a function of duration of exposure to 10 $\mu g\ ml^{-1}$ of NDI at $pH_e$ 7.0 **a**, or $pH_e$ 6.0 **b**, at a cell concentration of $10^5$ (diamonds), $10^6$ (triangles) or $10^7$ (squares) cells $ml^{-1}$. Mean and range of triplicate plates are indicated. The results shown are typical of those obtained in three separate experiments.

### *Effect of cell concentration on toxicity of NDI*

Previous experiments with NDI which were performed in monolayer culture demonstrated decreased toxicity as confluency was approached (Miller *et al.*, 1983; Wilson *et al.*, 1987). We therefore carried out experiments to determine the toxicity of NDI as a function of cell concentration. For both cell lines, cell killing diminished as the concentration of cells increased (Figure 3a). The toxicity of NDI showed only small differences for suspensions containing $10^5$ or $10^6$ cells per ml, but was greatly reduced at $10^7$ cells per ml. At a cell concentration of $10^7\ ml^{-1}$, toxicity of NDI was observed only at doses greater than 30 $\mu g\ ml^{-1}$. Even at high cell concentration, cell killing remained dependent on $pH_e$ (compare Figure 3a and 3b).

A possible explanation for the loss of activity of NDI in the presence of a high concentration of cells may be the breakdown of the compound under these conditions. We were unable to measure the amount of NDI present using spectrophotometry, and therefore used a bioassay in order to determine whether or not ths was occurring. No loss of activity of NDI was demonstrated in these experiments (Figure 4).

An increase in the expression of P-glycoprotein with as cells approach confluency, combined with active drug efflux due to the activity of P-glycoprotein, has been proposed as the basis for the cell concentration effect of NDI (Wilson et al., 1991). In order to test whether this was a major mechanism we carried out experiments with the $CH^RC5$ cell line which overexpresses P-glycoprotein and is 180-fold resistant to colchicine, compared to parental AUX B1 cells (Juliano & Ling, 1976). Following treatment with NDI at either $pH_e$ 6.5 or 7.4, there was only a small difference in the survival of cells from this cell line when compared to its parental cell line AuxB1 (~1.5-fold resistance, Figure 5).

*Spheroids*

We studied the cytotoxic effects of NDI on EMT-6 spheroids at $pH_e$ 6.0 and 7.0. Following treatment of spheroids with NDI, there was no loss of cell yield after trypsinisation. Spheroids of diameter 500–600 µm were relatively resistant to cell killing by NDI although $pH_e$-dependent effects were still observed. Surviving fraction remained above 0.1 with concentrations of NDI up to 75 µg $ml^{-1}$ at $pH_e$ 7.0 and 50 µg $ml^{-1}$ at $pH_e$ 6.0 (Figure 6). At a concentration of 100 µg $ml^{-1}$ there was a marked decline in surviving fraction to $10^{-5}$. By contrast, when an equal number of spheroids were disaggregated prior to exposure and treated with NDI in a single cell suspension, surviving fraction was reduced considerably (Figure 6).

**Figure 6** Survival of EMT-6 cells, from spheroids which were treated with different concentrations of NDI for 4 h at $pH_e$ 6.0 (solid symbols) or 7.0 (open symbols). Spheroids were either treated intact (diamonds) or were dissociated first and then treated (squares). Mean and range of triplicate plates are shown from an individual experiment and are typical of the results obtained from three experiments.

**Figure 4** Results of bioassay experiments. EMT-6 cells ($10^7$ cells $ml^{-1}$) were treated with NDI (10 µg $ml^{-1}$) for 4 h, and plated after centrifugation. The supernatant was used to treat cells at a concentration of $10^5$ cells $ml^{-1}$. The column marked 'No prior exposure' represents survival of $10^5$ cells $ml^{-1}$ treated with NDI that had not been used to treat other cells previously. Columns represent data from a single experiment (mean and range of triplicate plates) and are typical of results from two experiments.

### *Effect of the cysteine protease inhibitor E-64 on toxicity of NDI*

Experiments were carried out in the presence of the cysteine protease inhibitor E-64 in order to determine the importance of these enzymes as mediators of cell killing by NDI. After 24 h incubation in the presence of E-64 (100 µg $ml^{-1}$), EMT-6 cells continued to grow normally, but had levels of cathepsin B + L and H that were reduced to < 1% and 30% of control, respectively (data not shown). Preincubation with E-64 produced some protection of EMT-6 cells against killing by NDI at $pH_e$ 7.0 but the effects of E-64 were not significant and considerable cell killing was observed in the presence of the inhibitor (Table I). The effects of E-64 were also assessed with low concentrations of NDI at $pH_e$ 6.0. Under these conditions, survival of cells pre-treated with E-64 was similar to that of cells treated with NDI alone (Table I). The failure of E-64 to prevent or markedly diminish the cytotoxicity of NDI, particularly at $pH_e$ 6.0, suggests that cysteine proteases such as the cathepsins do not play a major role in cell killing under these conditions.

**Figure 5** Survival of AUX B1 cells (squares) and $CH^RC5$ cells (diamonds) treated with NDI (10 µg $ml^{-1}$) at $pH_e$ 6.5 (solid symbols) or 7.4 (open symbols) for varying lengths of time. Mean and range of triplicate plates are shown. The results shown are typical of those obtained from three separate experiments.

**Table I** Effect of the cysteine protease inhibitor E-64 on cell survival following treatment with NDI at $pH_e$ 7.0 for 4 h or at $pH_e$ 6.0 for 1 h

| *Conditions* | *Surviving fraction* | |
|---|---|---|
| *$pH_e$ 7.0* (4 h) | | |
| NDI 5 µg $ml^{-1}$ | $6.0 \times 10^{-3}$ | $P = 0.60$ |
| NDI 5 µg $ml^{-1}$ + E-64 | $1.2 \times 10^{-2}$ | |
| NDI 10 µg $ml^{-1}$ | $3.2 \times 10^{-4}$ | $P = 0.46$ |
| NDI 10 µg $ml^{-1}$ + E-64 | $2.4 \times 10^{-3}$ | |
| *$pH_e$ 6.0* (1 h) | | |
| NDI 0.1 µg $ml^{-1}$ | $7.6 \times 10^{-1}$ | $P = 0.69$ |
| NDI 0.1 µg $ml^{-1}$ + E-64 | $7.5 \times 10^{-1}$ | |
| NDI 1 µg $ml^{-1}$ | $2.8 \times 10^{-1}$ | $P = 0.78$ |
| NDI 1 µg $ml^{-1}$ + E-64 | $3.0 \times 10^{-1}$ | |

Data shown are the mean of two experiments and are compared using Student's *t*-test.

*Erythrocyte lysis experiments*

An alternative mechanism by which a detergent such as NDI might exert its cytotoxic effect is by interaction with the cell membrane. In order to determine if this could occur at near-physiological pH, we assessed the effect of NDI on human erythrocytes suspended in a physiological saline solution. At $pH_e$ 7.4 NDI caused lysis of erythrocytes at concentrations of 10 $\mu g\ ml^{-1}$ and above (Table II). As the concentration of NDI was increased the extent of erythrocyte lysis also increased but never reached 100% (relative to the lysis caused by Triton X-100). At high concentrations (50–80 $\mu g\ ml^{-1}$) the degree of lysis decreased slightly. A similar pattern was observed in experiments carried out at pH 7.75. By contrast, at pH 6.0 and 6.6, complete lysis was observed, with no decrease in lysis as higher concentrations of NDI were used. There was no decrease in the minimum concentration of NDI required to cause lysis as a function of pH. A cell concentration effect, similar to that observed in the cell survival experiments, was also noted. At a concentration of approximately $10^7$ erythrocytes $ml^{-1}$, lysis was markedly decreased as compared to erythrocytes suspended at a concentration of $10^6$ cells $ml^{-1}$; a 4-fold increase in the concentration of NDI was required to produce equivalent lysis (data not shown). This effect was observed independent of $pH_e$. These observations suggested that the basis of the cell concentration effect may be inadequate amounts of a detergent acting on the cell surface membrane, in the face of increasing quantities of cell membrane.

*Effect of cell concentration on toxicity of other detergents*

We performed survival experiments with two other detergents known to act on the cell surface membrane (Triton X-100 and sodium dodecyl sulphate (SDS)) in order to determine if a cell concentration effect was a general property of compounds that disrupted the plasma membrane. Survival was at undetectable levels ($<10^{-5}$) when cells, at a density of $10^5$ or $10^6$ cells $ml^{-1}$, were exposed to 0.0125% Triton X-100 for 4 h. Cells at a density of $10^7$ cells $ml^{-1}$ exposed to the same conditions had a surviving fraction of 0.1. Similar results were obtained in experiments using SDS (Figure 7), suggesting that decreased cell killing at high cell concentration may be a general property of membrane disrupting detergents. However, reduced levels of $pH_e$ did not enhance the cytotoxicity of either of these detergents (Figure 7).

### Discussion

The present experiments show that NDI kills cells in a $pH_e$ dependent manner. Toxicity was not prevented by an inhibitor of cysteine protease (E-64) suggesting only a minor role for these lysosomal enzymes in mediating the toxicity of NDI. Toxicity of NDI was markedly dependent on cell concentration, as was true for other detergents.

The $pH_e$ dependent toxicity of NDI occurs over the range of values of $pH_e$ that may exist in some regions of solid tumours. One hundred-fold greater cell killing occurred at $pH_e$ 6.6 compared to 7.4. Toxicity was even greater at levels of $pH_e$ below 6.6, though measurements of $pH_e$ in tumours have only rarely revealed values as low as this (Wike-Hooley *et al.*, 1984). The therapeutic potential of NDI is limited, however, by its loss of activity at high cell concentrations. The concentration of cells within a solid tumour would be expected to be even higher than the highest concentration used in our experiments ($10^7$ cells $ml^{-1}$). Furthermore, the results of our spheroid experiments suggest poor therapeutic efficacy of this compound in solid tissue. This may be due to high cell concentration, to limited penetration of NDI in tissue and/or to limited access to cell membranes.

Previous studies of the effects of NDI have demonstrated that it is toxic to cultured cells, when viability is assessed by the endpoint of LDH release or failure to exclude dyes from cells. Several lines of evidence suggested that the cytotoxicity of NDI was mediated by release of lysosomal enzymes (Miller *et al.*, 1983; Wilson *et al.*, 1987; Wilson *et al.*, 1989). Firtst, NDI or similar detergents, at concentrations toxic to cultured mouse peritoneal macrophages, were not able to cause lysis of erythrocytes, which lack lysosomes (Firestone & Pisano, 1979). Secondly, the cysteine protease inhibitor E-64, afforded cells considerable protection from the effects of NDI. After incubation with 100 $\mu g\ ml^{-1}$ of E-64, the activity of cathepsins B and L in Chinese hamster ovary fibroblasts was reduced to 19% of control values; these cells were almost completely resistant to the effects of NDI (Wilson *et al.*, 1987). Finally, experiments carried out on human fibroblasts from patients with I-cell disease, which have levels of lysosomal hydrolases that are 10–15% of normal (Heismann & Herschkowitz, 1974; Neufeld *et al.*, 1975), revealed a marked reduction in the cytotoxicity of NDI (Wilson *et al.*, 1987).

In contrast, other investigators have suggested that NDI is unable to disrupt the lysosomal membrane. Forster *et al.* (1987) studied the effects of NDI and N-dodecylmorpholine (another lysosomotropic detergent) on isolated lysosomes and intact cells. They used four different techniques in an attempt to demonstrate an increase in the permeability of the lysosomal membrane after exposure to NDI or N-dodecylmorpholine but were unable to detect any change in permeability to either small or large molecules.

Our results suggest that NDI does not exert its cytotoxic effect solely via an action on lysosomes. At concentrations similar to those causing cell death in cultured cell lines (as measured by colony-forming ability) we observed lysis of erythrocytes. Since mature human erythrocytes lack lysosomes, a mechanism of action which does not involve these organelles must be operative. The discrepancy between our results, and those previously reported by Firestone and

**Figure 7** Survival of EMT-6 cells following treatment with 0.01% SDS for 1 h at $pH_e$ 7.0 (open squares) or 6.0 (solid squares). Cells were suspended at different concentrations during treatment with the detergent. Mean and range of triplicate plates are indicated and are typical of the results obtained in two experiments.

**Table II** Erythrocyte lysis caused by exposure to varying concentrations of NDI for 5 min

| *Concentration of NDI ($\mu g\ ml^{-1}$)* | *Extracellular pH* 6.0 | 6.6 | 7.4 | 7.75 |
|---|---|---|---|---|
| 0 | <1 | <1 | <1 | <1 |
| 10 | 41 | 60 | 79 | 32 |
| 20 | 94 | 101 | 88 | 91 |
| 30 | 99 | 99 | 88 | 88 |
| 40 | 100 | 104 | 81 | 86 |
| 50 | 99 | 102 | 78 | 72 |
| 80 | | 100 | 58 | |

Results are expressed as a proportion of the absorbance of cells treated with 0.1% Triton X-100 and are the means of three experiments.

Pisano (1979) who were unable to detect erythrocyte lysis with another of the acid activated detergents may be explained partially by the concentration of red cells used. In our experiments, we used $10^6$, $10^7$ or $10^8$ erythrocytes $ml^{-1}$, and noted decreasing sensitivity to the effects of NDI as cell concentration increased. At lower values of $pH_e$ (6.0, 6.6) greater lysis of erythrocytes was observed at higher concentrations of NDI ($\geqslant 20\ \mu g\ ml^{-1}$), which could be the result of a greater proportion of the extracellular detergent being in the active protonated form under these conditions. However, we were unable to demonstrate lysis with reduced concentrations of NDI at low $pH_e$ (as compared to $pH_e$ 7.0–7.4). In the earlier report, investigators used a concentration of red cells of $3.5 \times 10^7$ cells $ml^{-1}$ and a powerful acid activated detergent. The combination of these factors may have resulted in the negative finding.

On the basis of the protective effect observed with the cysteine protease inhibitor E-64 in previous experiments, cathepsins were proposed as the major toxic enzymes which mediated cell killing by NDI (Wilson *et al.*, 1987). We found that E-64 led to only a minor protective effect against cell killing, at concentrations which inhibited cathepsins B + L and H. A possible explanation for the discrepancy in results may be the different endpoints used. Wilson *et al.* (1987) used LDH release as an indicator of cell death, and observed a reduction in release after pretreatment of cells with E-64. In our experiments, clonogenic survival was used, which is a more relevant endpoint for cell survival if the goal is to inhibit the reproductive potential of tumour cells. Our findings suggest that in the cell lines tested by us, cysteine proteases do not play a major part in the cytotoxicity of NDI. However. lysosomes contain many other enzymes and it is possible that they may contribute to the observed toxicity. The availability of specific inhibitors of these enzymes could help to clarify what part, if any, they play in the toxicity of NDI, but few such inhibitors are available.

An alternative mechanism by which NDI may exert its cytotoxic effect is by direct damage to the plasma membrane. In order for this to take place, sufficient NDI must exist in the protonated (active detergent) form outside the cell. Although the acid activated detergents were designed so that they would only become active detergents at reduced pH, a proportion of the compounds exist in the protonated form, even at near-physiological pH. For NDI, with a $pK_a$ of 6.3, approximately 17% of the compound is in the protonated or active form at pH 7.0. It is possible that this may result in a sufficient amount of detergent being present to damage the plasma membrane from the outside of the cell. As $pH_e$ is lowered, a larger proportion of the compound is in this form and the $pH_e$ dependent cell killing might be explained by an increase in extracellular concentration of detergent. A consequence of increased extracellular protonation of NDI is that less of the compound will be able to enter cells. The marked increase in toxicity noted at $pH_e$ 6.0 is thus more likely to be mediated by a mechanism that does not require entry of NDI into the cell. The results of our experiments with erythrocytes also support a mechanism of action which involves the action of NDI on the plasma membrane from the outside of the cell.

The effects of detergents on biological membranes are concentration dependent. At low concentrations, detergent molecules bind to the membrane. This may result in solubilisation of membrane proteins without changes to the overall structure of the membrane (Kagawa, 1972, Coleman, 1973). At higher concentrations, complete solubilisation of the membrane may occur. The extent of solubilisation is dependent mainly on the amount of detergent bound relative to the amount of membrane present (Helenius & Simons, 1975). The ratio of detergent to membrane lipid that is required for solubilisation is known for some common detergents, although the values (weight/weight) are only approximate; for Triton X-100 it is 1.9, while for SDS it is 1.6 (Helenius & Simons, 1975). Thus the ability of detergents to cause cellular lysis is likely to decrease with increasing cell number.

Previous investigations into the toxicity of NDI, carried out on monolayers of cells, have noted decreased activity as confluency was approached (Miller *et al.*, 1983; Wilson *et al.*, 1989). Changes in lysosomal number (per cell) or activity of lysosomal enzymes as cells approach confluency appear to be minor and do not explain the effect (Williams *et al.*, 1973; Wilson *et al.*, 1989). We also noted resistance to the cytotoxicity of NDI as the concentration of cells in suspension increased. These findings are consistent with an effect of NDI on the plasma membrane. Increasing cell concentration is associated with a large increase in the amount of membrane lipids present. Under these conditions, there may be insufficient NDI present to result in membrane solubilisation. Two detergents known to dissolve the cell plasma membrane, Triton X-100 and SDS, were also tested and were found to demonstrate a similar dependence on cell density; a marked decrease in cytotoxicity was seen as cell density increased. Furthermore, we noted a similar effect of cell concentration when testing the ability of NDI to cause lysis of erythrocytes.

An alternate explanation for the loss of toxicity of NDI as cells approach confluency has been proposed. Activity of the 170 kD membrane P-glycoprotein, which has been implicated in the multidrug resistant phenotype, has been reported to be greater in cells that were approaching confluence than in those in an exponential growth phase (Wilson *et al.*, 1991). The toxic effects of NDI were decreased in confluent cells, but could be enhanced by the calcium antagonists nifedipine and verapamil which can inhibit the drug efflux activity of P-glycoprotein. It was thus suggested that extrusion of NDI by P-glycoprotein was the basis of the loss of activity of NDI (as measured by LDH release) in confluent cells. However, using the same cell lines as Wilson *et al.*, we were unable to detect any difference in the cytotoxicity of NDI (as measured by clonogenic assay) betwen the P-glycoprotein overexpressing cell line $CH^RC5$ and its parent. The different endpoints used to measure cytotoxicity could account for this discrepancy. Furthermore, in our cell concentration experiments we studied the effects of NDI against cells which were growing exponentially, and which were then resuspended at varying cell concentration; the mechanism proposed by Wilson *et al.* (1991) cannot explain the cell concentration-dependent efects of NDI toxicity observed in our experiments.

We have shown that NDI is toxic to malignant cells in culture and that this toxicity is $pH_e$ dependent. Although agents with $pH_e$ dependent toxicity have considerable potential for causing selective toxicity in solid tumours, the marked loss of activity with increasing cell concentration and poor activity in spheroids suggests limited potential for NDI to be a useful anticancer agent.

### References

BARRETT, A.J. & KIRSCHKE, H. (1981). Assay of cathepsin H and cathepsin L. *Methods Enzymol.*, **80**, 539–541.

BARRETT, A.J., KEMBHAVI, A.A., BROWN, M.A., KIRSCHKE, H., KNIGHT, C.G., TAMAI, M. & HANADA, K. (1982). L-trans-Epoxysuccinyl-leucylamido(4-guanidino)butane (E-64) and its analogues as inhibitors of cysteine proteinases including cathepsins B, H and L. *Biochem. J.*, **201**, 189–198.

COLEMAN, R. (1973). Membrane bound enzymes and membrane ultrastructure. *Biochim. Biophys. Acta*, **300**, 1–30.

FIRESTONE, R.A. & PISANO, J.M. (1979). Lysosomotropic agents. 1. Synthesis and cytotoxic action of lysosomotropic detergents. *J. Med. Chem.*, **22**, 1130–1133.

FORSTER, S., SCARLETT, L. & LLOYD, J.B. (1987). The effect of lysosomotropic detergents on the permeability properties of the lysosome membrane. *Biochim. Biophys. Acta*, **924**, 452–457.

HEISMANN, U.N. & HERSCHKOWITZ, N.N. (1974). Studies on the pathogenic mechanism of I-cell disease in cultured fibroblasts. *Pediatr. Res.*, **8**, 865–870.

HELENIUS, A. & SIMONS, K. (1975). Solubilization of membranes by detergents. *Biochim. Biophys. Acta*, **415**, 29–79.

JULIANO, R.L. & LING, V. (1976). A surface glycoprotein modulating drug permeability in Chinese hamster ovary cell mutants. *Biochim. Biophys. Acta*, **455**, 152–162.

KAGAWA, Y. (1972). Reconstitution of oxidative phosphorylation. *Biochim. Biophys. Acta*, **265**, 297–338.

MILLER, D.K., GRIFFITHS, E., LENARD, J. & FIRSTONE, R.A. (1983). Cell killing by lysosomotropic detergents. *J. Cell. Biol.*, **97**, 1841–1851.

MOHINDRA, J.K. & RAUTH, A.M. (1976). Increased cell killing by metronidazole and nitrofurazone of hypoxic compared to aerobic mammalian cells. *Cancer Res.*, **36**, 930–936.

NEUFELD, E.G., LIM, T.W. & SHAPIRO, L.J. (1975). Inherited disorders of lysosomal metabolism. *Ann. Rev. Biochem.*, **44**, 357–376.

SUTHERLAND, R.M. (1988). Cell and environment interactions in tumour microregions: the multicell spheroid model. *Science*, **240**, 177–184.

TANNOCK, I.F. & ROTIN, D. (1989). Acid pH in tumors and its potential for therapeutic exploitation. *Cancer Res.*, **49**, 4373–4384.

WIKE-HOOLEY, J.L., HAVEMAN, J. & REINHOLD, J.S. (1984). The relevance of tumor pH to the treatment of malignant disease. *Radiother. Oncol.*, **2**, 343–366.

WILLIAMS, G.M., STROMBERG, K. & KROES, R. (1973). Cytochemical and ultrastructural alterations associated with confluent growth in cell cultures of epithelial-like cells from rat liver. *Lab. Invest.*, **29**, 293–303.

WILSON, P.D., FIRESTONE, R.A. & LENARD J. (1987). The role of lysosomal enzymes in killing of mammalian cells by the lysosomotropic detergent N-dodecylimidazole. *J. Cell. Biol.*, **104**, 1223–1229.

WILSON, P.D., HRENIUK, D. & LENARD, J. (1989). Reduced cytotoxicity of the lysosomotropic detergent N-dodecylimidazole after differentiation of HL60 promyelocytes. *Cancer Res.*, **49**, 507–510.

WILSON, P.D., HRENIUK, D. & LENARD, J. (1991). A relationship between multidrug resistance and growth-state dependent cytotoxicity of the lysosomotropic detergent N-dodecylimidazole. *Biochem. Biophys. Res. Commun.*, **176**, 1377–1382.